# The Advantage of Proton Therapy in Hypothalamic-Pituitary Axis and Hippocampus Avoidance for Children with Medulloblastoma

**DOI:** 10.14338/IJPT-21-00001.1

**Published:** 2021-08-02

**Authors:** Saif Aljabab, Shushan Rana, Shadonna Maes, Avril O'Ryan-Blair, Jackie Castro, Jack Zheng, Lia M. Halasz, Phillip J. Taddei

**Affiliations:** 1 Radiation Oncology Department, King Saud University, Riyadh, Saudi Arabia; 2 Radiation Oncology Department, University of Washington School of Medicine, Seattle, WA, USA; 3 Seattle Cancer Care Alliance Proton Therapy Center, Seattle, WA, USA; 4 Radiation Medicine Program, the Ottawa Hospital Cancer Centre, Ottawa, ON, Canada; 5 Department of Radiation Oncology, Mayo Clinic, Rochester, MN, USA

**Keywords:** craniospinal irradiation, hippocampus avoidance, pituitary avoidance, IMPT, VMAT, tomotherapy

## Abstract

**Purpose:**

Craniospinal irradiation (CSI) improves clinical outcomes at the cost of long-term neuroendocrine and cognitive sequelae. The purpose of this pilot study was to determine whether hypothalamic-pituitary axis (HPA) and hippocampus avoidance (HPA-HA) with intensity-modulated proton therapy (IMPT) can potentially reduce this morbidity compared with standard x-ray CSI.

**Materials and Methods:**

We retrospectively evaluated 10 patients with medulloblastoma (mean, 7 years; range, 4-14 years). Target volumes and organs at risk were delineated as per our local protocol and the ACNS0331 atlas. An experienced neuroradiologist verified the HPA and hippocampus contours. The primary objective was CSI and boost clinical target volume (CTV) covering 95% of the volume (D_95_) > 99% coverage with robustness. Described proton therapy doses in grays are prescribed using a biological effectiveness relative to photon therapy of 1.1. The combined prescribed dose in the boost target was 54 Gy. Secondary objectives included the HPA and hippocampus composite average dose (D_mean_ ≤ 18 Gy). For each patient, volumetric modulated arc radiotherapy (VMAT) and tomotherapy (TOMO) plans existed previously, and a new plan was generated with 3 cranial and 1 or 2 spinal beams for pencil-beam scanning delivery. Statistical comparison was performed with 1-way analysis of variance.

**Results:**

Compared with standard CSI, HPA-HA CSI had statistically significant decreases in the composite doses received by the HPA (32.2 versus 17.9 Gy; *P* < .001) and hippocampi (39.8 versus 22.8 Gy; *P* < .001). The composite HPA D_mean_ was lower in IMPT plans (17.9 Gy) compared with that of VMAT (21.8 Gy) and TOMO (21.2 Gy) plans (*P* = .05). Hippocampi composite D_mean_ was also lower in IMPT plans (21 Gy) compared with that of VMAT (27.5 Gy) and TOMO (27.2 Gy) plans (*P* = .02). The IMPT CTV D_95_ coverage was lower in IMPT plans (52.8 Gy) compared with that of VMAT (54.6 Gy) and TOMO (54.6 Gy) plans (*P* < .001) The spared mean volume was only 1.35% (19.8 cm^3^) of the whole-brain CTV volume (1476 cm^3^).

**Conclusion:**

We found that IMPT has the strong potential to reduce the dose to the HPA and hippocampus, compared with standard x-ray CSI while maintaining target coverage. A prospective clinical trial is required to establish the safety, efficacy, and toxicity of this novel CSI approach.

## Introduction

As the most-common pediatric brain cancer, medulloblastoma has witnessed several treatment advances beyond initial cytoreductive surgery. However, the high propensity of subarachnoid dissemination necessitates craniospinal irradiation (CSI) in addition to postoperative posterior fossa (PF) radiation for local regional control [1]. Historically, CSI was delivered to 36 Gy, followed by a PF boost of 18 to 19.8 Gy without systemic therapy. However, that came at the expense of neuroendocrine and cognitive toxicities that resulted in long-term decreased quality of life (QOL) [2, 3].

For standard-risk medulloblastoma, the incorporation of concurrent and adjuvant systemic therapy has enabled craniospinal dose reduction with the preservation of therapeutic efficacy [4, 5]. A further attempt at CSI dose reduction was implemented in the Children's Oncology Group ACNS0331 clinical trial [6], which randomized 3 to seven-year-old patients with standard-risk medulloblastoma to 18 Gy versus 23.4 Gy. Although neurocognitive results remain pending, disappointingly, CSI dose reduction was associated with worse event-free survival and overall survival [7].

Chief among the central nervous system (CNS) substructures affected by CSI are the hypothalamic-pituitary axes (HPA) and the hippocampi. Endocrine toxicity after CSI remains prevalent, with an incidence of approximately 70% at 5 years and a median time to endocrinopathy diagnosis of 38 months [8]. The importance of hippocampal radiation-dose reduction, studied by Gondi et al [9] in adults with benign or low-grade tumors after fractionated stereotactic radiotherapy, has become more apparent. The recently published NRGCC001 trial [10] showed that patients with brain metastases randomized to hippocampal avoidance whole-brain radiation therapy (HA-WBRT) had better neurocognitive outcomes than did those who received standard WBRT.

Given these data, we performed an ad hoc dosimetric analysis on planning computed tomography scans from previously treated patients to describe the parameters and technical process required to reduce the hippocampi and HPA dose to < 18 Gy using intensity-modulated proton therapy (IMPT) and to maintain a boost prescription coverage of 23.4 Gy CSI in standard-risk medulloblastoma.

## Materials and Methods

### Preparation of Patient Data for Dosimetric Analysis

We obtained ethics approval and a confidential data-sharing agreement from both the University of Ottawa Ethics Board Committee and the University of Washington Institutional Review Board. We reviewed the data of 10 previously selected pediatric patients treated for embryonal CNS tumors. The study group (n = 10), with median age of 7 years (range, 4-14 years), had previously undergone maximal safe resection. Seven patients had midline tumors, and 3 patients had left lateralized lesions. Pathologic diagnosis confirmed medulloblastoma in 9 patients, whereas 1 patient was diagnosed with an atypical teratoid rhabdoid tumor. Six patients were simulated in the supine position and 4 patients in the prone position.

The original plans (simulation computed tomography images, fused magnetic resonance images, and original contoured structure sets) were electronically transferred to the Seattle Cancer Care Alliance Proton Therapy Center (Settle, Washington).

Targets, organs at risk (OARs), and their dose constraints were defined as per our study protocol (**Table 1**). Some additional structures not listed in **Table 1** included OARs with no specific dose constraint (as low as reasonable achievable), such as the lens, parotid gland, submandibular gland, breast (female only), larynx, mandible, stomach, spleen, and bladder. Optimization and planning structures, such as planning OAR volumes and volume of interest (VOI) for the HPA and hippocampi were also included. Original contours followed the ACNS0331 atlas, as previously described [11]. No modifications were made to the original-structure set contours or planned dose constraints, except for the added brain stem constraint of V_54_ < 5 cm^3^. This objective was added to accommodate the change in IMPT planning practice because of concerns of increased risk of brain-stem necrosis versus photon therapy [12–14].

**Table 1.** Structure set and dose constraints for intensity-modulated proton therapy (IMPT) craniospinal irradiation (CSI) plans.

**Structure**	**Dose prescription and constraint**
Targets	
Spinal canal	
CTVsp_23.4	
CTVwb_23.4	
PTV_CSI_23.4_5 mm	D_95_ ≥ 100% (23.4 Gy in 13 fractions)
GTV + surgical bed	
CTV_54_15 mm	
PTV_54_3 mm	D_95_ ≥ 100% (30.6 Gy in 17 fractions)
Organs at risk	
Supratentorial brain	V_54_ Gy < 5 cm^3^
HPA	D_mean_ ≤ 18 Gy
Hippocampi	D_mean_ ≤ 18 Gy
Brainstem	V_54_ Gy < 5 cm^3^
Optic nerves	D_0.03_ (cm^3^) < 54 Gy
Chiasm	D_0.03_ (cm^3^) < 54 Gy
Eyes	D_max_ < 45 Gy
Retinas	D_max_ < 45 Gy
Cochleas	D_mean_ < 45 Gy
Spinal cord	D_max_ ≤ 50 Gy
Thyroid	D_mean_ < 20 Gy
Heart	D_mean_ < 8 Gy
	V_15_ < 5%
	V_5_ < 70%
Lungs	D_mean_ < 9 Gy
	V_20_ < 10%
	V_5_ < 60%
Esophagus	D_mean_ < 20 Gy
Liver	D_mean_ < 10 Gy
	V_15_ < 15%
	V_5_ < 75%
Kidneys	D_mean_ < 7 Gy
	V_15_ < 5%
Small-bowel bag	D_mean_ < 12 Gy
	V_10_ < 65%

**Abbreviations:** CTV, clinical target volume; PTV, planning target volume; GTV, gross target volume; HPA, hypothalamic pituitary axis.

### IMPT Planning Technique

In this study, we describe only the IMPT HPA-HA CSI technique because VMAT and helical TOMO techniques have been previously described [11]. A US board-certified dosimetrist and medical physicist, both of whom specialize in proton therapy, performed the planning and verification. A clinically commissioned treatment planning system (TPS; version 6, RaySearch Laboratories AB, Stockholm, Sweden) was used for inverse treatment planning and robust optimization [15]. Proton therapy doses in grays were prescribed using a biological effectiveness relative (RBE) to photon therapy of 1.1.

We used the same beam arrangements for all IMPT plans. Each patient's plan comprised posterior-anterior fields to target the thecal sac and 2 posterior oblique fields with a third posterior field to target the brain. The left posterior oblique field angles varied between 120° and 160°, whereas the right posterior oblique fields ranged from 200° to 240°. The subsequent boost-plan field arrangement was defined according to varying patient-specific CTV geometry. Beam angles included those mentioned previously for the initial plan.

Following the International Commission on Radiation Units and Measurements' recommendation [16], we applied an RBE value of 1.1 for protons, such that the clinical goal dose of 18 Gy corresponded to an absorbed dose of 16.4 Gy. The largest snout was 40 cm longitudinally, which permitted a sufficient treatment length of 36 cm. We separated the targets into 3 optimization structures: brain, upper spine, and lower spine. Following our institution's standard of practice for IMPT, these volumes deliberately overlapped by 6 cm, with a gradual gradient to ensure a firmly robust dose distribution within the target volumes. The inferior aspect of the brain optimization target was 2 cm or more superior to the shoulders. We used these structures for spot placement during optimization.

After creating the spot placeholders, we cropped the hippocampi 5-mm VOI and the HPA 5-mm VOI from the CTV brain target. The VOI volumes directed the TPS optimizer to avoid spot placement in that section, which allowed for a sharp dose falloff and a minimal dose to those VOIs. The planning target volumes (PTVs) that overlapped with the HPA or hippocampi were not cropped from those structures.

We used 2 posterior oblique beams because they better spared the lens and covered the cribriform plate compared with 2 lateral beams. We also routinely added a third posterior field as it improved conformality, coverage, robustness analysis, and structure avoidance. Vertex and superior anterior oblique beams were avoided because matching them with the superior spinal field was deemed insufficiently robust.

We used a pencil-beam algorithm for dose scoring and spot fluence weighting with automatic scaling values of 0.9 for energy-layer spacing, 0.8 for hexagonal-spot spacing, and 0.5 for lateral-target margins [17]. For robust optimization, we evaluated the plan by varying the Hounsfield units by ± 3% range and with 3-mm geometric shifts in 6 directions (± x, ± y, and ± z). We applied an independent-beam function to the TPS to allow for robust optimization of individual beams. We then performed quality assurance (QA) on each of the fields, comparing the TPS-calculated dose in a 2-dimensional plane to the response of a 2-dimensional ionization chamber array (Matrixx PT, IBA, Louvain-La-Neuve, Belgium). Because most of our physics field-QA test pass, we performed the standard-of-practice field-QA for 1 plan only, which passed with > 90% γ-index at 3% dose-difference and 3-mm distance to agreement.

**Figure 1.**
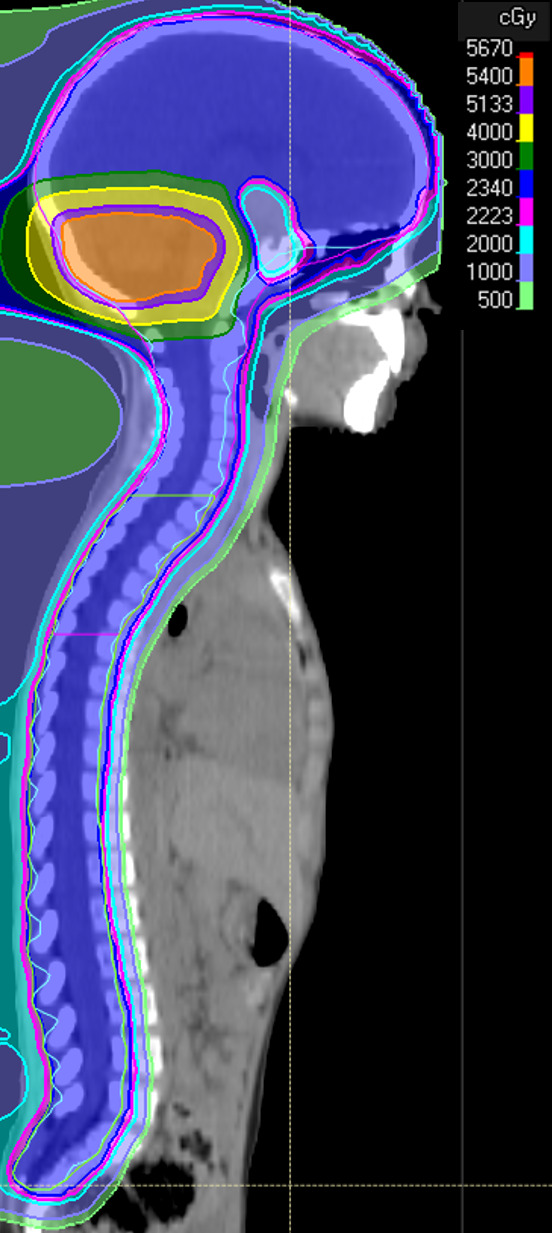
Sagittal view of the intensity-modulated proton therapy (IMPT) hypothalamic-pituitary axis and hippocampus sparing craniospinal irradiation (CSI) plan.

The objectives and constraints for IMPT inverse planning were set in the following manner. The primary objective was to keep the brain stem V_54_ < 5 cm^3^. The secondary objective was to achieve a CTV D_95_ > 99% with robustness in a Boolean subtraction of the 5-mm VOI from the PTV. The third objective was to keep the composite mean dose in the HPA and hippocampus to ≤ 18 Gy. Each plan was optimized using full multifield optimization and > 200 robustness scenarios [18]. A sagittal view of the final product for 1 patient can be seen in **Figure 1**.

### Dosimetric and Statistical Analysis

We performed statistical analysis with the SPSS statistics (version 23, IBM, Armonk, New York) and Prism (version 8, GraphPad Software, La Jolla, California) software. The statistical significance threshold for the 1-way analysis of variance (ANOVA) test was set to α = 0.05. We analyzed all plans based on characteristics in coverage and dose avoidance of the HPA and hippocampi. The CTV coverage was chosen, instead of PTV, because of institutional differences and the absence of robustness testing with intensity-modulated radiation therapy (IMRT) plans. Robust optimization for the CTV was set to a lower dose covering 95% and an upper dose received by 2% of the volume. For HPA and HAs, the mean dose was the primary metric used. Comparison of indices between IMRT and IMPT (ie, homogeneity index, conformality index, and gradient index) was not performed because proton therapy dosimetry is inherently different than x-ray therapy dosimetry, and the interpretation of such data would be challenging. The dosimetric data for all other contoured OARs were collected and analyzed.

## Results

### Target Coverage

Regarding different HPA-HA CSI techniques, IMPT composite CTV D_95_ coverage was lower in IMPT plans (97.8%) compared with VMAT (100%) and TOMO (100%) plans (*P* < 0.001). The slightly lower coverage is due to the additional IMPT brain stem constraint of V_54_ < 5 cm^3^. Similarly, the D_95_ composite-PTV coverage was lower for IMPT (94%) compared with both VMAT (100%) and TOMO (100%). However, the CSI PTV D_95_ coverage was similar between all 3 (98.4%, 98.4%, and 98%, *P* = .86). The average composite CTV hot spots (D2) for IMPT (55.7 Gy, 103%) were comparable with TOMO (55.6 Gy, 103%), and both were slightly less than VMAT (56.6 Gy, 105%) plans (*P* < .01).

On average, HPA-HA CSI IMPT plans achieved a slightly lower coverage in the 23.4-Gy CTV compared with the standard nonsparing CSI IMPT plan (D_95_, 97.3% versus 100%, *P* < .01) as well as the 54-Gy CTV (D_95_, 97.8% versus 98.9%, *P* = .12). Composite CTV hot spots were comparable between both modalities, despite statistical significance (D_2_, 55.7 Gy versus 55.3 Gy, *P* = .01).

### OARs Avoidance

**Figure 2.**
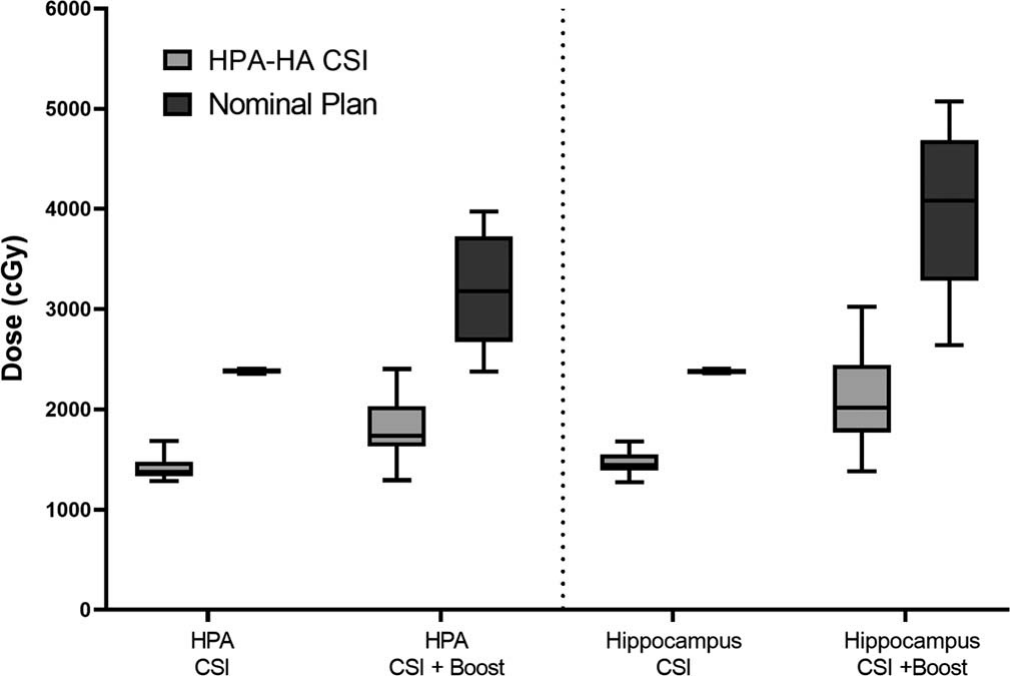
Plot comparing hypothalamic-pituitary axis (HPA) and hippocampi doses with nominal versus HPA–hippocampus avoidance (HA) craniospinal irradiation (CSI) plans.

**Figure 3.**
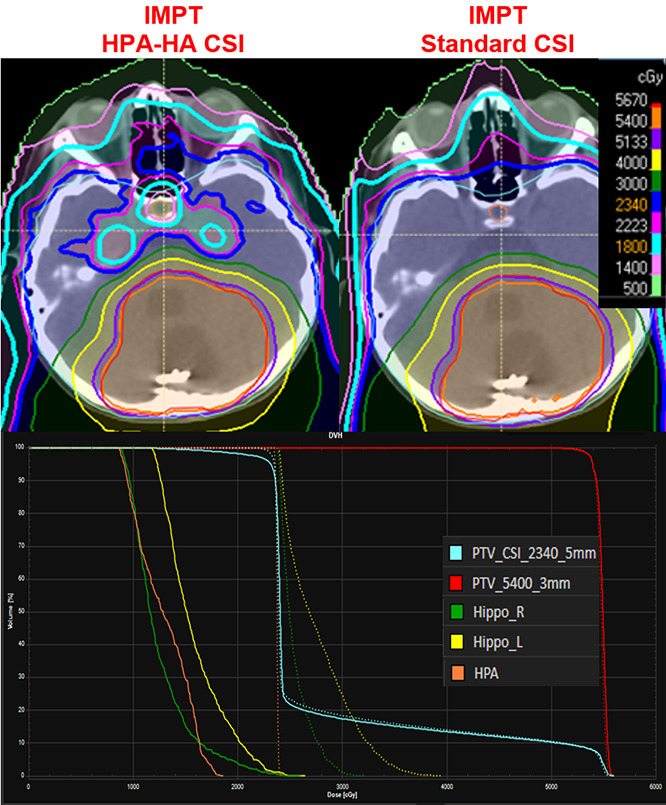
Comparative plan and dose-volume histogram (DVH) between intensity-modulated proton therapy (IMPT) hypothalamic-pituitary axis and hippocampus avoidance (HPA-HA) sparing craniospinal irradiation (CSI; left, dose-volume histogram [DVH] solid line) and standard CSI (right, DVH dotted line).

The IMPT HPA-HA CSI plans significantly reduced the HPA and hippocampi dose compared with standard CSI with IMPT (**Table 2** and **Figure 2**). The HPA composite D_mean_ was reduced from 32.2 Gy to 17.9 Gy (*P* < .001) and the composite hippocampi D_mean_ from 39.8 Gy to 21 Gy (*P* < .001). The plans and dose-volume histogram (DVH) of these 2 modalities are shown in **Figure 3**.

**Table 2.** Dosimetric comparison between intensity-modulated proton therapy (IMPT) standard craniospinal irradiation (S-CSI) and hypothalamic-pituitary axis and hippocampus avoidance (HPA-HA) CSI and other HPA-HA CSI techniques (n = 10).

**Dosimetric comparison between S-CSI and HPA-HA IMPT CSI**				
**Parameter**	**IMPT S-CSI** **(Gy or Gy-RBE)**	**IMPT HPA-HA CSI (Gy or Gy-RBE)**		***P*** **value, 1-way ANOVA**
HPA D_mean_	23.8	14.1		< .001
Composite^a^ HPA D_mean_	32.2	17.9		< .001
Hippocampi D_mean_	23.8	14.7		.01
Composite^a^ hippocampi D_mean_	39.8	21		< .001
**HPA-HA CSI dosimetric comparison between VMAT versus TOMO versus IMPT**				
**Parameter (Gy or Gy-RBE)**	**VMAT**	**TOMO**	**IMPT**	***P*** **value, 1-way ANOVA**
HPA D_mean_	13.9	15	14.1	.11
Composite^a^ HPA D_mean_	21.8	21.2	17.9	.05
Hippocampi D_mean_	17.2	15.9	14.7	< .01
Composite^a^ hippocampi D_mean_	27.5	27.2	21	.02

**Abbreviations:** RBE, radiobiological effectiveness; ANOVA, analysis of variance; VMAT, volumetric modulated arc radiotherapy; TOMO, tomotherapy.

a Composite: initial CSI phase dose contribution plus the boost-phase dose contribution.

**Figure 4.**
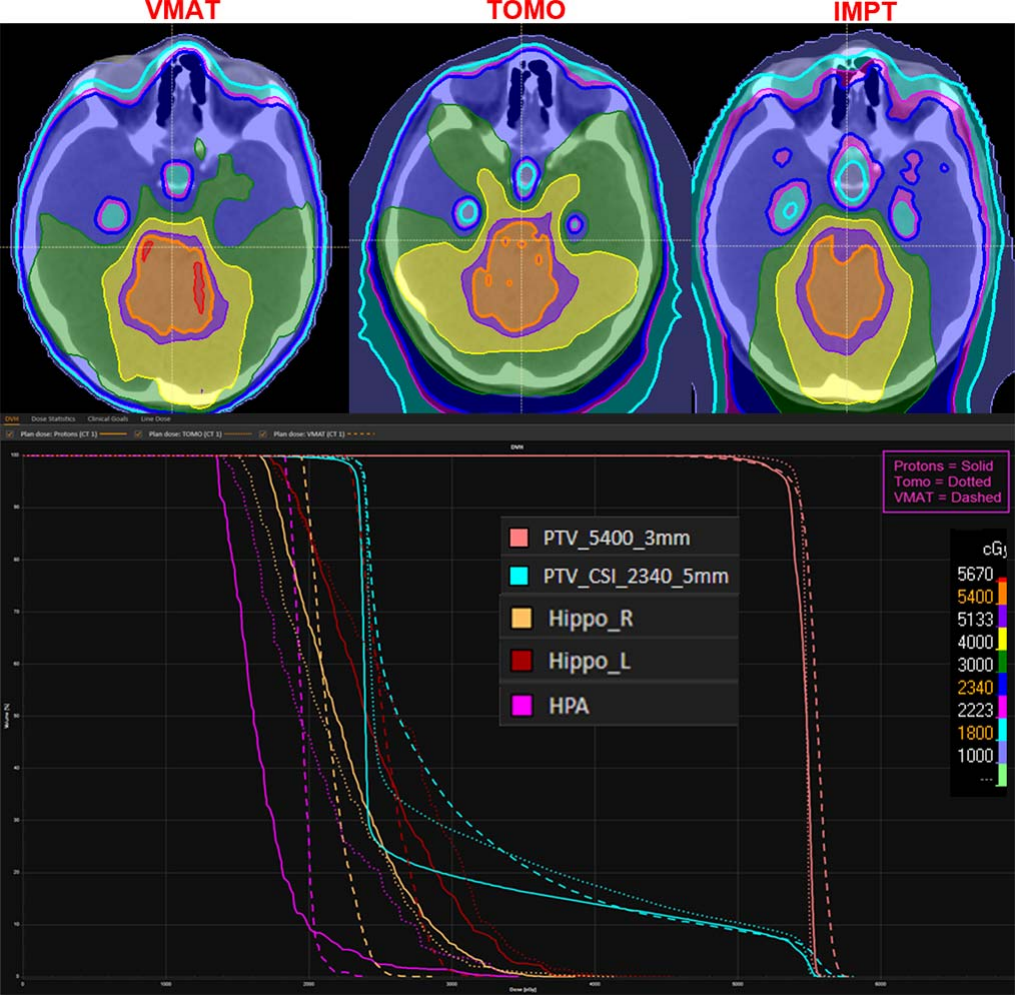
Comparative plan and dose-volume histogram (DVH) for hypothalamic-pituitary axis and hippocampus avoidance (HPA-HA) craniospinal irradiation (CSI) volumetric modulated arc radiotherapy (VMAT) versus tomotherapy (TOMO) versus intensity-modulated proton therapy (IMPT).

Comparing the different HPA-HA CSI techniques, during the CSI phase, IMPT successfully achieved a mean dose < 18 Gy to the HPA (14.1 Gy) and hippocampi (14.7 Gy). With the boost's addition, the HPA objective was not compromised with a final mean dose of 17.9 Gy. However, the hippocampi did receive an additional 6.3 Gy with a final mean dose of 21.0 Gy. Similarly, for VMAT and TOMO, the HPA (13.9 Gy and 15 Gy, respectively) and hippocampi (17.2 Gy and 15.9 Gy, respectively) constraints were met during the CSI phase. However, the final dose for both structures exceeded 21 Gy during the boost phase (**Table 2**). Axial views and DVHs of these 3 modalities for 1 patient are shown in **Figure 4**.

For other OARs, IMPT achieved the most cumulative-dose constraints compared with VMAT and TOMO. The IMPT was less likely to exceed the cochlear mean dose and the cervical cord maximum dose constraints. As prioritized, all IMPT plans achieved brainstem V_54_ < 5 cm^3^, whereas VMAT and TOMO achieved that in 2 patients. Additionally, it was difficult to achieve suitable lung and heart constraints for the VMAT plans, with values of V_5_ reaching 71% and 80%, respectively. Both modalities met all other OAR constraints.

## Discussion

In this study, we implemented, to our knowledge, the first technical and dosimetric feasibility analysis of HA and HPA using IMPT in CSI. Our results illustrate the capabilities of modern IMPT techniques. We were able to produce acceptable CSI plans and avoid important intracranial structures. This technique can reduce the dose received by the HPA-HA by > 50%. Consequently, this can significantly improve patient QOL, especially in the long term. In this study, we outlined the technical details to produce such a plan.

The decline in neurologic function is a prevalent long-term side effect of radiation therapy in adult and pediatric populations. It is widely accepted that this decline is associated proportionately with radiation dose to CNS substructures. For instance, higher hippocampal dose has been associated with decrements in multiple domains, including motor speed, dexterity, and short-term and long-term recall [19, 20]. Merchant et al [21] prospectively followed children treated with craniospinal irradiation and demonstrated a significant association between radiation dose to specific brain substructures and longitudinal cognitive scores. They reported that age and mean radiation dose to the temporal lobes and hippocampi had significant effect on longitudinal scores. Compounding the neurocognitive decline is the endocrine dysfunction associated with higher hypothalamus and pituitary doses [22].

With advancements in radiation therapy capabilities, HA has correspondingly evolved to mitigate the cognitive decline observed in HA-WBRT. The RTOG 0933 clinical trial [23] provided initial prospective data on this technique in patients with brain metastases treated with WBRT to 30 Gy in 10 fractions and reported that conformal avoidance of the hippocampus was associated with memory preservation and improved QOL as compared with historical series. The NRG CC001 trial [10] further expanded on HA-WBRT versus WBRT in a phase III study. The study reported significantly lower risk of cognitive failure, mainly attributable to less deterioration in executive function at 4 months (23.3% versus 40.4%, *P* = .01), than at 6 months in learning (11.5% versus 24.7%, *P* = .049) and at 6 months in memory (16.4% versus 33.3.%, *P* = .02). The HA-WBRT was also more tolerable, with fewer reported adverse events and no difference in survival outcomes.

It is reasonable to extrapolate that these cognitive benefits would be at a larger magnitude for younger patients receiving higher doses of radiotherapy, such as in our cohort. Beyond photon-based techniques, proton therapy's unique spatial properties have recently garnered increased attention in HA. Stoker et al [24] performed an IMPT dosimetry analysis comparing HA IMPT versus VMAT in 10 pediatric patients previously treated with WBRT for high-grade embryonal tumors to 36 Gy in 20 fractions. The IMPT was found to decrease the mean hippocampal dose from 13.7 Gy seen in VMAT to 5.4 Gy [24, 25].

The after mentioned study highlights IMPT's ability to reduce the mean hippocampi dose further. However, we would like to point out a few key differences in our CSI approach. First, whole-brain coverage and brain-stem constraints were a priority in our approach, which is why we reduced the hippocampi planning OAR volume to a lesser extent (5 mm versus 8 mm) than with WBRT. Second, craniocaudal fields were avoided because they were insufficiently robust when matching with a superior spinal field. Our field arrangement might have limited our ability to spare the HPA-HA further, but it is currently more clinically applicable.

Hypothalamic-pituitary irradiation avoidance combined with HA was first proposed by Fan et al [26] in the setting of prophylactic cranial irradiation for limited-stage small cell lung cancer. Thirteen patients were treated with an equispaced coplanar IMRT plan, which preserved target coverage (D_100%_ > 95%) and limited the mean dose to both the HPA (10.7-11.1 Gy) and hippocampi (9.6 Gy) [26]. This approach could significantly reduce long-term effects if successfully applied to current radiation regimens of childhood CNS cancer. Hence, we used that combination with IMPT of medulloblastomas treated with CSI followed by intracranial boost.

Our dose constraints to both the hippocampus and HPA were derived from several clinical studies. In the case of the HPA, earlier attempts at 18 Gy CSI in medulloblastoma were associated with increased height and fewer endocrinopathies compared with matched controls who received the current standard-dose regimens [27]. The most extensive prospective series of children and young adults who received proton radiation found that the 5-year incidence of manifesting any pituitary hormone deficiency was 56% and 87%, with mean HPA dose > 20 Gy equivalent (GyE) and 40 GyE, respectively. Interestingly, a mean dose < 20 Gy was associated with a 9% incidence in growth hormone deficiency [22].

Regarding the hippocampus, RTOG 0933 [23] set dose constraints of D_100%_ < 9 Gy with a maximum dose of 16 Gy; however, that was with a WBRT regimen of 30 Gy in 10 fractions. Several neurocognitive data for childhood survivor studies suggested that a dose of 18 Gy is beneficial as compared with 24 Gy [28, 29]. Our study aimed to reduce the hippocampi dose to as low as possible (≤ 18 Gy) without affecting the CSI and boost coverage. For this study, we chose doses currently used for treatment standard-risk medulloblastoma; however, it could be that this technique is even more beneficial in the treatment of high-risk medulloblastoma given the greater risk of neurocognitive toxicity after 36 Gy.

We previously explored the use of HPA-HA CSI with VMAT and TOMO [11]. We could limit the total mean dose to both the HPA and hippocampi to ≤ 18 Gy during the CSI phase but not during the boost phase (**Table 2**). The IMPT, however, was consistently successful in meeting the HPA composite-dose constraints. Hippocampi composite mean doses exceeded 18 Gy with IMPT but were still lower than VMAT and TOMO plans (21 versus 27.5 versus 27.2, respectively).

Because of the decreased-dose contribution from the boost phase, improved HPA-HA with IMPT is possible. However, according to routine clinical practice, creating the IMPT plans with an additional brainstem constraint might have contributed to a slightly biased advantage in avoiding the HPA-HA compared with IMRT. The location and volume of the boost dictate which hippocampus is better spared. In some cases, both are equally spared, but in others, only one is effectively spared. It might be more feasible to focus on avoiding one hippocampus versus both, but its implications are unknown.

There remains a theoretical risk of increased disease recurrence in the proposed spared regions in dose-limitation strategies. However, unlike the ACNS0331 study [6], which reduced the entire brain dose to 18 Gy, we attempted to reduce the dose to only a small brain volume (< 2%). A recurrence within a 5-mm region surrounding the hippocampus has been defined in some retrospective brain metastases studies [30]. Perihippocampal involvement of brain metastases is estimated at approximately 10%, whereas proper hippocampus metastases are less common at 0% to 2%. Similar data in patients with medulloblastoma are limited to a post hoc analysis of 51 patients with high-risk medulloblastoma enrolled in the French trial [31]. Although 16% of patients with M+ disease at diagnosis (n = 28) had metastases within the first 5 mm of the hippocampus, those with M0 disease did not experience hippocampal region failure after 36 Gy CSI.

Regarding HPA involvement, there are limited data in brain-metastases literature. Janssen et al [32] analyzed an extensive series of 4280 metastases from 865 patients and found the rate of hypothalamic and pituitary metastases to be 0.6% and 0.2%, respectively. Because the available data are so limited, there is still much uncertainty regarding recurrence risks and the risk-to-benefit ratio. What recurrence risk is considered acceptable by clinicians and patients to mitigate long-term neurotoxicity? Should avoidance techniques only be explored with very low-risk medulloblastoma, such as the subgroup with WNT tumors? Should we attempt to spare only the HPA or only the hippocampi? These inquiries should be considered before designing a prospective trial.

Aside from ascertaining local control, other limitations in this study are secondary to its retrospective nature. Our analysis was limited to 10 patients, decreasing the power to detect dosimetric differences. Compared with our CSI technique, the optimal arrangement of beams remains to be determined because there is variability in IMPT HA across institutions [24]. Although multiple noncoplanar and coplanar beams could be used, that would theoretically add considerable treatment time, potential risk of prolonged anesthesia, and risk of positional displacement between fields, depending on image-guidance capabilities.

In conclusion, we found that IMPT has the strong potential to reduce the dose to the HPA and hippocampus compared with standard x-ray CSI and to maintain target coverage. A prospective clinical trial is required to establish the safety, efficacy, and toxicity of this novel CSI approach. Work remains to minimize the increased dose secondary to the boost field. Until then, this study does not provide the clinical evidence for adopting HPA-HA CSI; rather, it demonstrates its feasibility and suggests further evaluation in a prospective clinical trial.
